# Model-Driven Experimentation: A New Approach to Understand Mechanisms of Tertiary Lymphoid Tissue Formation, Function, and Therapeutic Resolution

**DOI:** 10.3389/fimmu.2016.00658

**Published:** 2017-04-04

**Authors:** James A. Butler, Jason Cosgrove, Kieran Alden, Jon Timmis, Mark Christopher Coles

**Affiliations:** ^1^Centre for Immunology and Infection, Department of Biology, Hull York Medical School, York, UK; ^2^Department of Electronics, University of York, York, UK; ^3^York Computational Immunology Laboratory, University of York, York, UK

**Keywords:** multi-scale modeling, tertiary lymphoid tissue, systems immunology, mechanistic modelling, model-driven experimentation

## Abstract

The molecular and cellular processes driving the formation of secondary lymphoid tissues have been extensively studied using a combination of mouse knockouts, lineage-specific reporter mice, gene expression analysis, immunohistochemistry, and flow cytometry. However, the mechanisms driving the formation and function of tertiary lymphoid tissue (TLT) experimental techniques have proven to be more enigmatic and controversial due to differences between experimental models and human disease pathology. Systems-based approaches including data-driven biological network analysis (gene interaction network, metabolic pathway network, cell–cell signaling, and cascade networks) and mechanistic modeling afford a novel perspective from which to understand TLT formation and identify mechanisms that may lead to the resolution of tissue pathology. In this perspective, we make the case for applying model-driven experimentation using two case studies, which combined simulations with experiments to identify mechanisms driving lymphoid tissue formation and function, and then discuss potential applications of this experimental paradigm to identify novel therapeutic targets for TLT pathology.

## Formation and Function of Secondary and Tertiary Immune Microenvironments

Lymphoid tissues are responsible for the orchestration of functional immune responses. This is achieved through the development and maintenance of niches that support the retention, activation, and proliferation of adaptive immune cells in response to antigenic stimulation. Adult lymphoid tissue architecture is organized by an underlying network of stromal cells that produce extracellular matrix (e.g., collagens) and provide survival (e.g., BAFF, IL-7), migratory (CCL19/21, CXCL13), and immune activation [the storage and presentation of immune complexes by follicular dendritic cell (FDC)] signals (1). Distinct stromal subsets with unique secretion profiles (chemokines, other cytokines, and survival factors) develop in response to signaling from lymphocytes with a key role for TNF superfamily receptors; this stromal–lymphocyte cross talk ensures the correct cell type is stimulated (or regulated) at the right time and place. Sustained cross talk between mesenchymal stroma and lymphocyte subsets is a core feature of lymphoid tissue formation and maintenance and occurs irrespective of the tissue type or anatomical location.

Formation of lymphoid tissues can occur by different cellular and molecular mechanisms. During fetal development, secondary lymphoid tissues form in a process dependent on the RAR-related orphan receptor gamma transcription factor expressing lymphoid tissue inducer cells (LTi) responding to localized chemotactic gradients leading to the formation of lymph nodes (LN) and Peyer’s patches (PPs) in a lymphotoxin β (LTβ)-dependent process (2). Localized mesenchyme, lymphoid tissue organizer (LTo) cells differentiate into adult marginal reticular cells, fibroblastic reticular cells, and FDCs (3). Likewise, in the adult, innate lymphoid cells type 3, the adult equivalent of LTi cells, have a key role in regulating cryptopatches that can mature into isolated lymphoid follicles (4). These specialized lymphoid structures contain predominantly B cells and often contain germinal center (GC) reactions.

In humans, tertiary lymphoid tissues (TLTs) are found in inflammatory immune responses associated with chronic pathology from hip joint replacements, keloids, tissues in autoimmune disease (e.g., the salivary gland in Sjogren’s syndrome, multiple sclerosis, and rheumatoid arthritis) to solid tumors and follicular lymphomas in the bone marrow (5–9). Although the role of specific cell types has been controversial, there is an emerging paradigm of a multistep process where localized inflammation induces stromal cell activation in a lymphocyte independent process, leading to localized microenvironments permissive for T and B cells entry (10). These lymphocytes have the potential to drive the formation of organized tertiary tissue in an autocrine-dependent process. This process closely resembles the capacity of naive B cells to drive B cell follicle formation in secondary lymphoid tissues in a TNFα- and LTβ-dependent process and the capacity of activated B cells to generate the GC, a transient microenvironment that drives high-affinity immune responses in a self-regulating autocrine-dependent process. In both secondary immune tissues (LN, PPs, and spleen) and TLTs including ILFs and TLT, activated B cells prime the formation of the GC reaction. This specialized microenvironment contains both activated and proliferating B cells and different stromal compartments of CXCL12-secreting stroma (dark zone) and CXCL13-secreting FDCs (light zone). This facilitates the cyclic selection and expansion of antigen-specific B cells (11).

Non-lymphoid inflammatory immune structures, granulomas, can form in the liver, intestine, adipose tissue (crown-like structures), and lung induced by chronic infection/inflammation associated with tuberculosis, sarcoidosis leishmaniasis, schistosomiasis, cell death, and Crohn’s disease (12–14). The formation of these highly dynamic microenvironments superficially resembles TLT; however, their formation and organization is driven by activated macrophages rather than by the mesenchymal–lymphocyte cross talk observed in lymphoid tissues thus do not exhibit lymphocyte compartmentalization. Granuloma structures are very heterogeneous in presentation within individual patients in a continuum between early macrophage centric granulomas, self-resolving granulomas, and fibroblastic structures, these often being fibrotic rather than taking on a supportive stromal network phenotype. The triggers that drive granuloma formation instead of TLT formation appear not to be due to differences in the different chemotactic cues delivered by activated macrophages compared to those delivered by activated stromal fibroblasts, leading to a very different cellular make up to the inflammatory foci of leukocytes [primarily myelomonocytic (granuloma) vs. lymphocytic (TLT)].

## Current Approaches to Studying Lymphoid Tissue Formation: Limits, Challenges, and New Approaches

Experimental studies, principally performed in gene knockout, lineage-specific fluorescent protein, and Cre reporter mouse lines have contributed significant insights into the roles of multiple different cell types and molecules in lymphoid tissue formation and function. This has been further validated using histology and flow cytometry analysis on human secondary lymphoid tissues. However, in contrast to secondary lymphoid tissues, there are some distinct differences in human tissue pathologies to those found in mice including the cellular composition of TLTs, granulomas, and other inflammatory tissues. This arises in part from genetic and physiological differences between human and mice including the timing and duration of the immune response (chronic vs. acute inflammation), the inflammatory triggers (infection, autoimmunity, and cancer), and transcriptional differences in immune cells in the different species. In general, mouse models of immune-mediated inflammatory disease are acute and fail to replicate the chronic human disease characterized by disease flairs followed by remission, limiting their translational capacity to human disease. Infection and tumor models in mice either rapidly resolve (too quickly for chronic pathology to establish) or lead to the mouse having to by euthanized for health and welfare prior to tertiary lymphoid pathology occurring. In comparison, humans may live the rest of their life with the disease pathology, particularly in the context of treatment with biologics and small molecules; thus, pathology has the opportunity to evolve from localized inflammation to fibrotic tissue failure, systemic inflammation, and autoimmunity working together to prevent disease resolution. Increasingly, human 3-dimensional tissue culture models containing both stroma and lymphocytes have become increasingly common and useful in understanding underlying molecule mechanisms of TLT formation. However, it is not currently possible to represent the full complexity of chronic human pathology *in vitro*.

Experimental systems (*in vivo* and *in vitro*) to date have proven limited in their ability to explain chronic clinical pathology and resolve established Sjogren’s pathology, although TNF has an important role in FDC differentiation and B cell organization, anti-TNF fails to induce resolution disease (15). To better understand the form and function of TLTs, current knowledge of stromal regulation through molecular signals and immune cell behavior within lymphoid tissue must be consolidated and considered in a quantitative, systems-based approach. The development of systems-level stochastic computational models can bring together a broad understanding across spatiotemporal scales of how genetic and molecular factors relate to cellular and tissue level form and function and give rise to the complex, functional architectures observed in secondary lymphoid organs and disease-specific TLTs. These models permit *in silico* experimentation providing a unique platform driving further experimentation and assessing novel mechanistic targets and intervention strategies where *in vivo* observed heterogeneity can be replicated.

Alan Turing (of code breaking fame) in seminal early work in mathematical biology (16) noted that gastrulation arose from symmetry breaking, and this leads to fundamental insights and principles that drive modern mathematical and computational biology: the notion that chaotic, non-linear behavior of individual biological processes, including the self-organization of complex biological structures (e.g., TLT), can result in emergent properties that cannot be understood from consideration of each individual component in isolation. The development of models that capture the essential, emergent behavior of specific biological processes, with extraneous components excluded, enables understanding of how complex molecular and cellular interactions govern complex, emergent biological processes and can therefore lead to new insights and quantitative predictions (17). Emergent properties in a TLT model would include stromal networks, lymphocyte organization, migration and interactions with antigen-presenting cells, and localized cytokine/chemokine production.

## Application of Model-Driven Experimentation (MDE) to Understand Mechanisms of Lymphoid Tissue Development and Function

Advances in computing resources and computational modeling technology have provided the capacity to generate complex *in silico* models of lymphoid tissues that incorporate space, time, and cellular heterogeneity found in immune tissues including TLT. Applying *in silico* approaches to understand secondary lymphoid tissue formation and function requires the integration of experimental data across cellular, molecular, and tissue levels of organization. Ensuring that the biological processes are appropriately described requires a fine balance between model abstraction and interpretation (quantitative and qualitative) of experimental data. A number of different modeling approaches may be utilized (summarized in Table 1), increasingly, integration of different mathematical/computational techniques into a hybrid model is a common strategy to address the limitations of using each technique in isolation. This approach also facilitates the consolidation of data across different levels of organization (molecular, cellular, tissue, and patient) into a single multi-scale model. For example, an agent-based model can capture an individual cell, which in turn incorporates a differential equation-based model capturing a “lower-level” aspect of that individual’s behavior, such as surface expression of a receptor (42). Adopting an *in silico* approach provides a platform that can provide insights and generate predictions that can be verified *in vivo*: verification that can lead to increased biological understanding and incrementally improved *in silico* models for further experimentation. This iterative approach of combining *in vivo, in vitro*, and *in silico* approaches has been termed “model-driven experimentation” (18).

**Table 1:** **Mathematical and computational techniques for modeling immune processes**.

Technique	Description	Comments
ODE	Ordinary differential equations: describe the rate of change with respect to one other variable (e.g., population change over time, *t*)	Commonly used technique that can be used to quantify changes in population size over time

PDE	Partial differential equations: describe rate of change of a function of more than one variable with respect to one of those variables (e.g., motion through space *x, y*, and *z* as a function of time *t*)	Often used to describe changes occurring over both time and multiple spatial dimensions

Monte Carlo	Statistical random sampling method where outcomes are determined at random from input probability distribution functions	Stochastic technique to model deterministic processes, very frequently integrated within ABM, CPM, and other stochastic modeling approaches

Petri nets	Graph-based model describing network of events or “transitions” that occur depending on given conditions or “places,” a stochastic methodology	Computationally efficient can be effectively defined using SBML2. Capturing explicit spatial representation can be difficult

ABMs	Agent-based models are composed of individual entities specified as agents, which exist independently in a well-defined state: a set of attributes at a specific point in, e.g., time and space, with state transitions governed by a rule-set, often described in terms of finite state machines and other diagrammatic constructs using the Unified Modeling Language	There are a number of methodologies to generate ABMs. There are tools with user interfaces for constructing simpler lattice-based ABMS or “unconstrained” models manually coded as software in languages such as Java and C++

(Extended) cellular Potts modeling	A lattice-based modeling technique for simulating the collective behavior of cells. A cell is defined as a set of pixels within a lattice (sharing a “spin state”) and is updated pixel-by-pixel according to a mathematical function, which incorporates cell volume and surface/adhesion energies	Similar to an ABM but relies on effective energy functions (the Hamiltonian) to describe cellular adhesion, signaling, motility, and other physical phenomena

Hybridized models	Bringing together a range of different techniques generally within the context of an ABM or CPM, incorporating differential equations and a variety of other mathematical and computational techniques to effectively capture phenomena occurring over different spatiotemporal scales (e.g., intracellular activity)	Can take advantage of different modeling techniques, particularly applicable where there are multiple processes occurring in different scales of time and space

## Case Study 1: Insights from MDE to Secondary Lymphoid Tissue Formation

Peyer’s patches are specialized secondary lymphoid tissues of the intestine that develop during a fixed window in fetal development and have an essential role in maintaining intestinal immunity. PPs form stochastically along the midgut, with mice developing 8–12 patches; however, as the absence of or reduction in the number of PPs is observed in several different gene knockouts, the molecular process that triggers patch formation was unclear (19). Using an MDE-based approach had the potential to provide new insight into how different signaling pathways (RET, chemokine receptors, cytokine receptors, TNF superfamily, and adhesion molecules) might integrate to induce PP development *in silico* and to subsequently design key experiments to test hypotheses *in vivo*. PPSim is an agent-based PP simulator that captures key processes during the 72-h period of tissue development in prenatal mice and replicates (statistically similar) emergent cell behaviors found *in vivo*, specifically populations of hematopoietic cells, known as lymphoid tissue initiator (LTin) and lymphoid tissue inducer (LTi) cells, migrates into the developing gut, with data from laboratory observations suggesting these cells follow a random motion. Both cell populations express receptors for the adhesion molecule VCAM-1, expressed by stromal LTo cells residing in the gut wall (20, 21). In this computational model, LTi and LTin are captured as individual entities that migrate into the developing midgut serosa and undergo a random walk, interacting with their localized simulated environment through signaling pathways including GDRFs/Ret signaling pathways, adhesion molecules, and chemokine receptors, as is observed *in vivo*. On ensuring PPsim adequately represented individual cell responses, statistical analysis techniques, specifically sensitivity analyses, were used to explore mechanisms driving prenatal lymphoid organ formation (22, 23). This exploration of the simulated biological pathways revealed which pathways had significant impacts on simulated cell behavior at different time points during PP development. By examining correlations in the level of activity of simulated pathways and cell behavior, the hypothesis was derived that contact between LTin and LTo cells that leads to the localized upregulation of VCAM-1 on stromal cells was the key triggering event that determined the site of PP formation on the midgut (21). Utilizing this prediction, an *in vitro* assay imaging fetal midgut explants incubated in the presence or absence of anti-VCAM-1 antibodies was developed. Using this assay, it was verified that early upregulation of VCAM-1 was the triggering event that was essential for the initiation of LTi and LTin cell clustering. The model simulation results, supported by replicated experimentation and safety-critical systems-based fitness-for-purpose argumentation that details the knowledge integration in model composition, provide evidence that the simulation was fit for the purpose of aiding exploration of this specific research question: understanding the triggering of lymphoid tissue development, which was not possible by conventional genetic approaches (24, 25).

## Case Study 2: Applying MDE to Understand GC Dynamics and Function

The GC reaction is a transient microenvironment in which affinity maturation occurs in response to immunization and infection, bearing key similarities to TLT in its evolution in the role of lymphocytes in inducing highly organized stromal networks, the essential role of TNF superfamily members in regulating its induction and the induction of chemokine gradients (10, 26). However, in comparison to TLT, the GC is a self-resolving tertiary lymphoid microenvironment. Recent technological advances, particularly the advent of intravital multiphoton imaging including photo-activated fluorescent proteins has led to the unprecedented availability of data on the dynamics B-cell migration and selection (27–30). However, imaging datasets provide a narrow window of insight into a process that occurs over a timescale of days and weeks. Furthermore, as imaging techniques are optimized for a given time and length scale, they are limited in their ability to link molecular, cellular, and tissue level processes. This has made the interpretation of imaging datasets in the context of the wider literature challenging. To address this issue, modeling approaches have been used to test the validity of different hypotheses of mechanisms controlling B-cell migration and selection within the GC (31–34).

In the GC reaction, model-derived insights have proved useful not only in the analysis of existing datasets but also as a driver for further experimentation. Specifically, an MDE approach has been used to examine the effects of antibody feedback on the process of affinity maturation (35). Analysis of an *in silico* GC reaction yielded the prediction that GC B-cells, which require antigen on FDCs for positive selection, were competing for antigen by early low-affinity antibodies. Only higher affinity B-cells were able to outcompete for antigen to receive the necessary survival signals. To experimentally validate this prediction, the authors manipulated the GC response with monoclonal antibodies of defined affinities and were able to confirm that antibody feedback provides a dynamic selection threshold to maximize Ig affinities (35). A similar approach was employed to investigate the role of toll-like receptor 4 (TLR4) on the GC where an iterative cycle of *in silico* and *in vivo* experimentation dissected the importance of TLR4 signaling on the maturation of FDCs, key regulators of B-cell selection in the light zone of the GC (36). Both of these MDE examples highlight the use of *in silico* experimentation as a means of refining experimental design through the identification of key time points and conditions to test *in vivo*. These case studies together provide example of how theoretical models can consolidate data from different sources as a platform for the development novel hypotheses and a driver for further experimentation.

## Perspective on MDE as Applied to TLT Formation, Function, and Therapeutic Resolution

**Figure 1:**
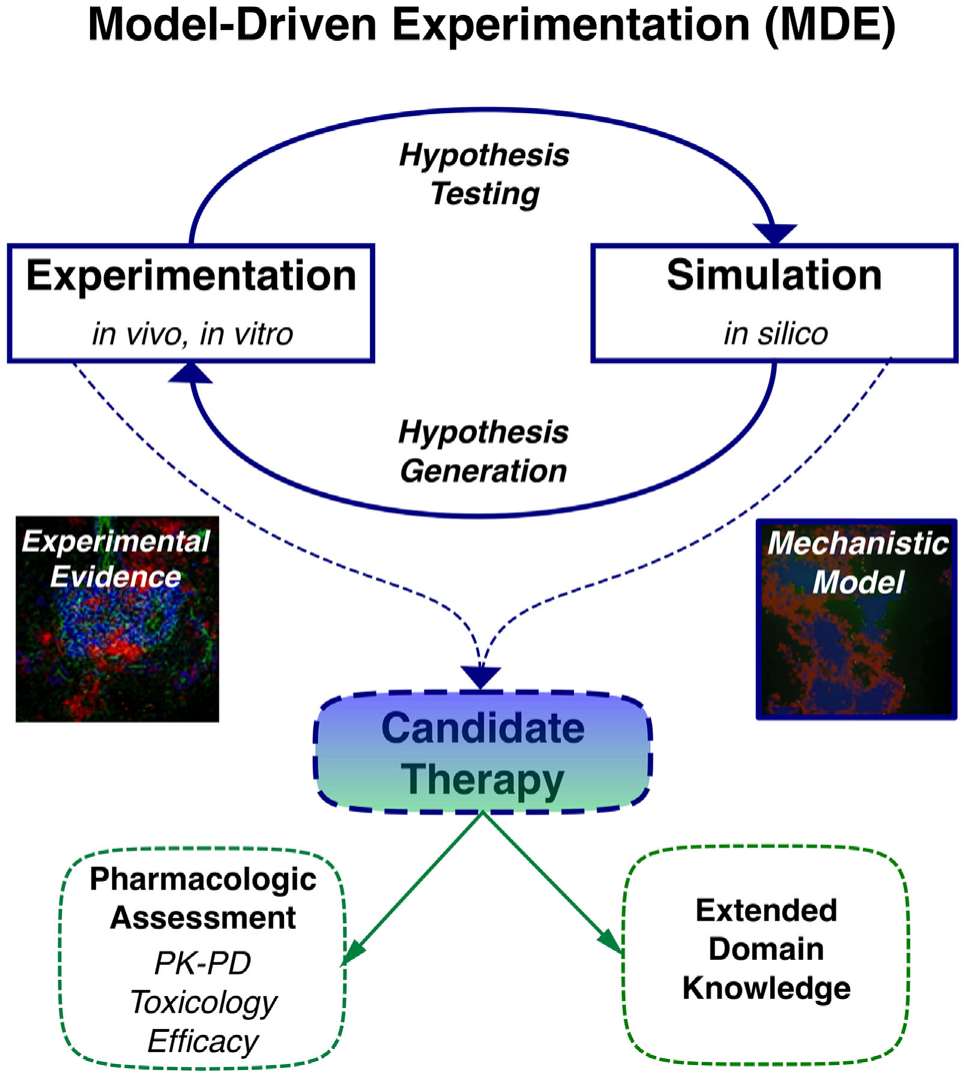
**Application of model-driven experimentation to develop new mechanistic understanding of tertiary lymphoid tissue (TLT) formation and maintenance permitting identification of novel therapeutic approaches to resolve localized TLT pathology**.

When computational modeling is combined with knowledge derived from imaging, multi-dimensional cytometry, and gene expression analysis of human TLT pathology, MDE has the potential to provide novel insights to key questions on molecular and cellular mechanisms involved in TLT formation, maintenance, and function similar to its capacity to impact on our understanding of lymphoid stromal network and granuloma dynamics (Table 2) (37–40). One of the key advantages of applying multi-scale modeling is it permits capture of a wide range of different phenomena that occur on different orders of magnitude in terms of time and length scales that are critical in the stochastic processes involved in TLT induction. These include different cell types, states and interactions, inflammatory molecules, extracellular matrix, adhesion molecules, and chemotactic signals all in the context of an evolving tissue microenvironment. Developing *in silico* models permits temporal inhibition of different signaling pathways and cellular depletions during different stages of TLT pathology using statistical tools (Figure 1). This permits identification of key pathways that could be targeted to induce resolution of pre-existing TLT rather than inhibiting its formation as has been used to make *in silico* predictions for the treatment of tuberculosis (41). A large number of novel antibody therapies, biologics, and small molecular inhibitors have been developed to target immune function for the treatment of immune-mediated inflammatory diseases. These therapies are unlikely to show maximal efficacy against existing tissue pathology when used as monotherapies, rather it is more likely that use of therapeutic combinations that is most likely to show clinical efficacy. The clinical challenge is that there are already over 20,000 possible different combinations using existing therapeutics that would need to be trialed to find optimal targeting strategy to resolve TLT pathology. Thus, MDE-based approaches provide a rational approach to identify novel combination therapeutic regimes that have a best potential in clinical trials (42).

**Table 2:** **Key questions on tertiary lymphoid tissue (TLT) formation and maintenance that can be address in hybridized TLT models**.

Formation
What are the minimum cellular requirements to initiate TLT formation? Is this driven by different types of stroma, lymphocytes, dendritic cells, or tissue-resident macrophage?
What is the relative importance of inflammation and antigen in TLT induction? Is autoantigen required for induction or just an outcome of the pathology?
What is the role of different cytokines and chemotactic signals on TLT formation?

**Maintenance**

What is the relative role of inflammatory cytokines, lymphocyte—stromal cross talk, immune cell entry, cell death, antigenic stimulation on TLT maintenance?
What are the key signaling pathways required to maintain TLT once it has formed? Can these pathways be targeted to induce TLT resolution?
Can TLT self-resolve in humans? If so, what is the balance between new TLT induction and resolution of existing structures?

Although the adoption of MDE has only recently started to impact on immunology research, it is starting to have a very significant impact on other areas of biology. We propose that the increased accessibility of computational models, the high-performance computing resources, the increased familiarity and understanding of simulations as tools to understand immune function, and the capacity to apply *in silico* approaches to identify potential therapeutic approaches and disease biomarkers will accelerate the application of MDE as a methodology understand and target disease resolution.

## Author Contributions

MC wrote this paper and is the corresponding author. JB, JC, KA and JT contributed to the writing of this perspective.

## Conflict of Interest Statement

The authors declare that the research was conducted in the absence of any commercial or financial relationships that could be construed as a potential conflict of interest.
